# Triglyceride-Rich Lipoproteins and Their Remnants as Silent Promoters of Atherosclerotic Cardiovascular Disease and Other Metabolic Disorders: A Review

**DOI:** 10.3390/nu13061774

**Published:** 2021-05-22

**Authors:** Radu Sascău, Alexandra Clement, Rodica Radu, Cristina Prisacariu, Cristian Stătescu

**Affiliations:** 1 Internal Medicine Department, ”Grigore T. Popa” University of Medicine and Pharmacy, 700115 Iași, Romania; radu.sascau@gmail.com (R.S.); rodiradu@hotmail.com (R.R.); cprisacariu88@gmail.com (C.P.); cstatescu@gmail.com (C.S.); 2 Cardiology Department, Institute of Cardiovascular Diseases Prof. Dr. George I.M. Georgescu, 700503 Iași, Romania

**Keywords:** triglyceride, triglyceride-rich lipoproteins, atherosclerosis, metabolic disorders, cardiovascular outcomes

## Abstract

While targeting elevated serum levels of low-density lipoprotein cholesterol has been the mainstay of atherosclerosis prevention and treatment for decades, the evidence regarding the atherogenic role of hypertriglyceridemia is still controversial. Various epidemiological population-based studies on statin-treated subjects nominated triglycerides, triglyceride-rich lipoproteins (namely, chylomicrons and very-low-density lipoprotein particles), and their remnants as major determinants of the substantial residual cardiovascular risk. With the triglyceride-glucose index and triglyceride to high-density lipoprotein ratio emerging as surrogate indicators of peripheral artery disease and atherosclerotic cerebrovascular disease, one can conclude that further research addressing the intricate relationship between triglycerides and atherosclerosis is warranted. Therefore, this review aims to provide insight into the current clinical and epidemiological state of knowledge on the relationship between triglycerides and atherosclerotic cardiovascular disease. It also intends to highlight the connection between triglycerides and other metabolic disorders, including diabetes mellitus, and the potential benefits of triglyceride-lowering agents on cardiovascular outcomes and all-cause mortality.

## 1. Epidemiological Background

Coronary artery disease (CHD), peripheral artery disease (PAD), and atherosclerotic cerebrovascular disease represent major consequences of atherosclerosis, with atherosclerosis-induced cardiovascular disease accounting for substantial clinical and economic burdens [1,2]. While targeting elevated serum levels of low-density lipoprotein cholesterol (LDL) has been the mainstay of atherosclerosis prevention and treatment for decades, the evidence regarding the atherogenic role of hypertriglyceridemia is still controversial [3].

Hypertriglyceridemia constitutes a common metabolic disorder characterized by elevated plasma concentration of triglycerides (TGs), greater than 150 mg/dL. Regarding its prevalence, a decreasing trend has been noticed for US adults aged 20 and above, from 33.3% during 2001–2004 to 25.1% during 2009–2012 [4].

Nevertheless, it must be acknowledged that the average of multiple TGs measurements possesses an incremental predictive power for cardiovascular events when compared to a single assessment. This information was validated by Aberra et al., who analyzed 15,792 individuals with no evidence of cardiovascular disease (CVD), from the Atherosclerosis Risk in Communities and Framingham Offspring studies, and followed up for 10 years. Notably, in this research, even “normal” or “optimal” concentrations of TGs (below 150 mg/dL) correlated with increased cardiovascular risk, particularly in women and in individuals with higher levels of high-density lipoprotein (HDL) [5].

Various epidemiological population-based studies on statin-treated subjects nominated TGs, triglyceride-rich lipoproteins (namely, chylomicrons and very-low-density lipoprotein particles (VLDL)), and their remnants as major determinants of the substantial residual cardiovascular risk [6]. This residual risk appears to be exceptionally high in subjects with type 2 diabetes mellitus (T2DM), metabolic syndrome, and obesity [7].

In 4988 Japanese diabetic statin-treated patients included in a posthoc analysis of the intEnsive statin therapy for hypercholesteroleMic Patients with diAbetic retinopathy (EMPATHY) study, levels of both fasting and non-fasting triglycerides (TGs) were positively linked to adverse cardiovascular events, thus emphasizing the significant residual cardiovascular risk attributable to TGs [8].

Regarding the implications of hypertriglyceridemia in the primary prevention of cardiovascular disease (CVD), a large Korean nationwide cohort study comprising 5,688,055 statin-naive young subjects, aged 20–39 years, demonstrated that from all four major lipid components (total cholesterol, LDL, HDL, and TGs), TGs concentration displayed the strongest correlation with the occurrence of death, myocardial infarction, and stroke (adjusted hazard ratio, 1.20; *p* < 0.001) [9].

Complementarily, the significance of preserving a normal lipid profile as the keystone of primary prevention of CVD has been reaffirmed by the results of a large retrospective observational study of 1,451,997 young adults, aged 20 to 49 years, from the Japan Medical Data Center health database. In this analysis, LDL ≥ 140 mg/dL, HDL < 40 mg/dL, and TGs ≥ 150 mg/dL were independently related to a higher incidence of myocardial infarction, angina pectoris, and heart failure during a median follow-up of 1148 ± 893 days [10].

In light of the above-mentioned data, this review aims to provide insight into the current clinical and epidemiological state of knowledge on the relationship between triglycerides and atherosclerotic cardiovascular disease. It also intends to highlight the connection between triglycerides and other metabolic disorders, including diabetes mellitus, and the potential benefits of triglyceride-lowering agents on cardiovascular outcomes and all-cause mortality.

## 2. Metabolism of Triglycerides, Triglyceride-Rich Lipoproteins, and Remnants

Triglyceride-rich lipoproteins (TRLs) is the generic term utilized for chylomicrons (CMs) and VLDL. Intermediate-density lipoprotein (IDL) particles are also indicated as TRLs, but their role has been less evaluated [11].

Exogenous TGs are absorbed by intestinal cells and incorporated in nascent CMs that are transported via the lymphatic system, wherein these particles are enriched with apolipoprotein C-II, apolipoprotein C-III, and apolipoprotein E. CM particles also incorporate apoB48, which is a surrogate of increased cardiometabolic risk. ApoB48 remnant lipoproteins result from CM lipolysis, and in accordance with the results of a cross-sectional study comprising 1045 young women and males (aged 17 years), increased fasting plasma apoB48 has been linked to a higher cardiometabolic risk in adolescents [12].

Endogenous TGs are mainly produced in the liver and secreted in the plasma via VLDL [13]. In hypertriglyceridemia, there is a marked elevation in the plasmatic levels of VLDL-VLDL_1_-particles (enclosing TGs in a proportion of 70% of the total mass), while the concentration of VLDL-VLDL_2_ (containing about 30% TGs) displays a moderate increase [14]. ApoB-100 is found only in VLD and IDL, is absent from CMs, and plays a pivotal role in atherogenesis, according to the most recent data that has reprioritized the role of TGs and TRLs in atherosclerotic cardiovascular disease (ASCVD) risk [11].

**Figure 1:**
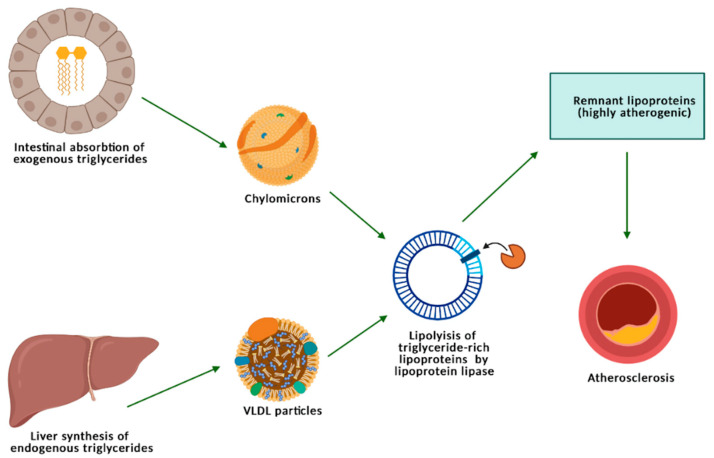
Metabolism of triglycerides, triglycerides-rich lipoproteins, and remnants.

TGs, located within the core of TRLs (CMs and VLDL) are hydrolyzed by lipoprotein lipase (to free fatty acids and glycerol), a metabolic process giving birth to the so-called “remnant” lipoproteins, which are highly atherogenic due to a high residual concentration of cholesterol, containing 40 times more cholesterol per particle than LDL (Figure 1) [15].

Although extensive epidemiological evidence links TGs, TRLs, and remnants to atherosclerotic cardiovascular disease, the underlying mechanistic relationship is still ambiguous and not fully understood. Apart from a larger cholesterol content of remnants, several other mechanisms have been hypothesized. Importantly, TRL remnants (also known as remnant cholesterol), due to their small size, are directly phagocytosed by macrophages to form foam cells, compared to LDL, which become atherogenic after being oxidized [16,17,18,19].

Interactions between apoB, apoE, and apoCIII enclosed in remnants and matrix proteoglycans favor the deposition of these particles within the arterial walls [20,21,22]. Oxidized free fatty acids resulting from TRL lipolysis trigger an important inflammatory response and alter endothelial function [6,23]. Concurrently, TRL remnants enhance the endothelial expression of proatherothrombogenic molecules (e.g., intercellular adhesion molecule-1, vascular cell adhesion molecule-1, and tissue factor), as emphasized by previous experimental studies, thus creating the optimal setting for their intimal retention [6,24]. In addition to endothelial dysfunction and increased production of proinflammatory molecules, TRLs remnants are accountable for the activation of the coagulation cascade and the inhibition of fibrinolysis [25,26].

On these bases, the association between TRLs’ remnants and increased cardiovascular risk has been supplementarily certified by a Danish study of 109,574 patients with a prior diagnosis of myocardial infarction or ischemic stroke, where a lower remnant cholesterol of 0.8 mmol L^−1^ in TRLs decreased the risk of major adverse cardiovascular events by 20% [27].

It is considered that TGs increase the risk of ASCVD via the VLDL-LDL delipidation process. This metabolic event results in enhanced amounts of LDL and remnant lipoproteins that exert a critical atherogenic effect on the arterial wall [14]. For this reason, the 2019 European Society of Cardiology and European Atherosclerosis Society guidelines in the management of dyslipidemias recommend statins as the first-line therapy in subjects with hypertriglyceridemia and high-risk of ASCVD [28].

Furthermore, TRL lipolysis came into view as the underlying explanation for the U-shaped relationship that has been broadly described between HDL-cholesterol levels and CVD risk. Large epidemiological studies proved that both reduced and increased concentrations of HDL are linked to a higher risk of CVD and mortality. Consequently, Feng et al. have hypothesized that during TRL lipolysis, a cholesterol transfer to HDL happens, which can possibly explain the atherogenic effect of high concentrations of HDL and the linkage between HDL and TGs metabolism [29].

As standard plasma TG concentration assessment does not offer any information regarding the levels or the structure of TG-rich lipoproteins, apolipoprotein B (apoB) measurement has emerged as a novel representative of hypertriglyceridemia. ApoB represents the main apolipoprotein component of both LDL and TG-rich lipoproteins and has already been appointed as a secondary treatment goal in subjects with high TG levels [30].

Apart from the established atherogenic role of apoB, TRLs, and remnants, polymorphisms in genes encoding for angiopoietin-related protein 3 (ANGPTL3) and apolipoprotein C-III (apoCIII) appear related to increased ASCVD risk. ANGPTL3 and apoCIII are involved in TGs and TRLs metabolism and act by inhibiting lipoprotein lipase.

Several pharmacological agents targeting apoCIII and ANGPTLIII are currently in phase II and III trials. In an analysis of the Atherosclerosis Risk in Communities (ARIC) study of 6359 participants followed up for 6 years, ANGPTL3 and apoCIII, along with TRL remnants and low-density lipoprotein triglycerides (LDL enriched with TGs), were significantly linked to CHD events. These results provided a research area that follows the potential benefits of lowering hepatic apoCIII and ANGPTL3 expression in patients with high concentrations of TRL [31].

## 3. The Association between Triglyceride-Rich Lipoproteins and Peripheral Artery Disease

PAD is one of the three main clinical manifestations of systemic atherosclerosis, along with atherosclerotic cerebrovascular disease and CHD, being particularly frequent in individuals aged 50 and above [32]. PAD accounts for substantial mortality and morbidity, and the identification of its major risk factors has always been a matter of great concern.

The REGICOR study was a prospective population-based analysis conducted in Girona province in northeastern Spain. It was the largest epidemiological study that sought to evaluate the incidence of peripheral vascular disease together with its main associated risk factors in individuals living in the Mediterranean area. The REGICOR study results signaled that the level of TGs was among the most important risk factors for PAD, together with smoking status, diabetes, age, history of CVD, and systolic blood pressure. Of note, the incidence of PAD recorded in the Mediterranean region appears to be lower than for other geographical zones [33].

Kou et al. applied Cox proportional hazard models in the Atherosclerosis Risk in Communities (ARIC) study cohort in order to describe the connection between conventional and novel lipid parameters and incident peripheral vascular disease. A total of 8330 individuals with no evidence of PAD at baseline were identified and included in the final analysis. During a median follow-up time of 17 years, 246 incident PAD cases were recorded. Higher baseline concentrations of triglycerides-related lipids and lower levels of HDL were independently linked to incident peripheral vascular disease [34].

Importantly, hypertriglyceridemia not only predicts the risk of PAD, but it also encompasses important therapeutic implications. According to an analysis of the Optum Research Database that contains the electronic health data of over 80 million individuals from the USA, among whom 1.6 million statin-treated and other 390,000 subjects meeting the inclusion criteria were identified, increased TGs concentration predicted the need for peripheral arterial revascularization. In patients aged 45 years and above with evidence of ASCVD and/or diabetes, the Kaplan-Meier analysis revealed that higher TGs levels resulted in an increased need for peripheral revascularization [35].

Over the years, other unconventional metabolic risk factors have also been affiliated with lower extremity arterial disease. In a cross-sectional analysis conducted on 10,900 Chinese hypertensive adults, nontraditional lipid profile indices were positively and independently associated with PAD in a dose-dependent manner [36].

Consequently, the atherogenic index or the triglyceride- to-HDL ratio and the triglyceride-glucose index have emerged as powerful predictors of ASCVD and, implicitly, of PAD. The triglyceride-glucose index is obtained via the formula Ln (fasting triglycerides (mg/dL) × fasting blood glucose (mg/dL)/2) and was used initially as a marker of insulin resistance in healthy individuals [37]. However, da Silva et al. underscored that this parameter is positively linked to a higher rate of symptomatic CHD and metabolic and behavioral risk factors [38]. Later on, the usability of the triglyceride-glucose index in clinical practice as a cost-effective marker of ASCVD was endorsed by the results of a large retrospective observational cohort study of 55,593,134 subjects aged 40 years and above included in the National Health Information Database of South Korea, where higher values of this index were connected to an increased risk of major complications of atherosclerosis [39].

Finally, a retrospective analysis that comprised a total of 71 subjects proved that the triglyceride-glucose index is an independent predictor of PAD severity. All these patients underwent peripheral angiography, and based on the severity of the lesions, the study population was segregated into two different groups. Group 1 included the individuals with type A and type B stenosis, according to the TransAtlantic InterSociety Consensus (TASC) II classification, while group 2 consisted of those with lesions of grade C and D. The triglyceride-glucose index appeared to be considerably higher in group 2 [40].

By using a similar study design, Mesut et al. demonstrated the association between triglycerides-to-HDL ratio or the atherogenic index and the severity of peripheral vascular disease, but on a larger scale, with a cohort of 412 patients who underwent angiographic evaluations. The study population was grouped on the basis of the TASC II classification too. In group 1 were included the subjects with TASC A-B lesions, while those with C-D lesions were assigned to group 2. Triglycerides and the triglyceride-to-HDL ratio were considerably higher in group 2 (*p* = 0.022 and *p* < 0.001) [41].

As previously mentioned, apoB containing lipoproteins (VLDL and CMs) became accountable for the elevated residual cardiovascular risk reported in subjects optimally treated with cholesterol-lowering agents. Nonetheless, in patients with clinical evidence of CVD, increased concentration of VLDL is a predictor more of future major adverse limb events than of subsequent major adverse cardiovascular events and all-cause mortality, as underscored by the study of Heidemann et al. In 8057 individuals with CVD from the Utrecht Cardiovascular Cohort–Secondary Manifestations of ARTerial disease (UCC-SMART) study, the association between major adverse limb events and plasma VLDL concentration was independent of other risk factors, including LDL and hypolipidemic medication [42].

Therefore, it is essential to assess TRLs levels in order to improve the detection of an enhanced risk of atherosclerotic events. A prospective case-cohort analysis within the Women’s Health Study identified that increased concentrations of TRLs correlate with a 2-fold increase in the risk of total CVD and incident coronary and cerebrovascular disease, a 3-fold increased risk of myocardial infarction, and a 2.5-fold increased risk of PAD. Notably, in this research, small density LDL was not associated with the development of peripheral vascular disease, thus highlighting the role TRLs play in the development of PAD [43].

## 4. The Linkage between Triglycerides and Atherosclerotic Cerebrovascular Disease

As stated earlier, a strong relationship between TGs and atherosclerotic cerebrovascular disease exists. A Mendelian randomization study of 514,791 patients from the MEGASTROKE consortium emphasized that higher plasma concentrations of apoB, LDL, and TGs resulted in a higher risk of any ischemic stroke, large artery stroke, and small vessel stroke. Notably, the multivariable Mendelian randomization analysis showed that apoB is the main factor responsible for the apoB, LDL, and TGs-related strokes (*p* < 0.005). Any ischemic stroke was the definition used for all types of stroke, except for intracerebral hemorrhage (large artery ischemic stroke, cardioembolic ischemic stroke, small vessel ischemic stroke, and ischemic stroke of undefined subtype) [44].

The triglyceride-to-HDL ratio and the triglyceride-glucose index also came to light as surrogates of carotid atherosclerosis and increased risk of ischemic stroke. Of note, in the literature, there is an inconsistent definition of the atherogenic index, expressed as either the triglyceride-to-HDL ratio or as the logarithmic transformation of the triglyceride-to-HDL ratio.

The triglyceride-glucose index is associated not only with atherosclerotic cerebrovascular disease but also with subclinical cerebral small vessel disease. In a multivariable logistic regression analysis of 2615 subjects’ health data by the Seoul National University Hospital Health Promotion Center, the triglyceride-glucose index was positively associated with the burden of silent brain infarcts, in a dose-response pattern (*p* for trend = 0.006). Higher triglyceride-glucose index values resulted in a greater prevalence of silent brain infarcts [45].

Additionally, the triglyceride-glucose index appears helpful in predicting the prognosis of patients with ischemic strokes. A total of 16,310 individuals who suffered from an ischemic stroke from the China National Stroke Registry II were included in a study that sought to evaluate the relationship between triglyceride-glucose index values and neurological outcomes. The multivariable Cox regression and logistic regression analyses indicated that the triglyceride-to-glucose index significantly correlated with the risk of stroke recurrence at 12 months, all-cause mortality, and neurological worsening in subjects with ischemic stroke [46].

Initially utilized as an indicator of insulin resistance, the triglyceride-to-HDL ratio describes a positive correlation with subclinical carotid atherosclerosis in postmenopausal women and with silent brain infarcts in men [47,48]. It appears valuable in predicting the risk of ASCVD and ischemic stroke events, as emphasized by the study of Zhou et al. on 9368 Chinese participants [49].

A single-center case-controlled study, including 31 patients with ischemic stroke due to symptomatic carotid artery stenosis and 236 subjects with ischemic infarctions not because of carotid artery stenosis, revealed that the logarithmic transformation of the ratio of the plasmatic TGs levels to HDL was the only lipid parameter to correlate with symptomatic carotid artery stenosis [50].

The atherogenic index of plasma also turned effective in predicting the occurrence of cerebrovascular accidents in patients with antineutrophil cytoplasmic antibody-associated vasculitis (AAV). One-hundred sixty-seven individuals, first diagnosed with AAV, and 300 healthy volunteers were recruited in the study of Ahn et al. Based on the logarithmic transformation of the TGs-to-HDL ratio cut-off value (0.11), the AAV patients were further divided into two groups. In the Kaplan Meyer analysis, it has been shown that AAV patients with an atherogenic index higher than 0.11 presented a significantly reduced rate of cerebrovascular accident–free survival when compared to those with an atherogenic index <0.11 (*p* = 0.027). Of note, higher atherogenic index values were recorded in the AAV group than in the control group (*p* < 0.001) [51].

## 5. An Insight into Other Implications of Hypertriglyceridemia, Beyond Atherosclerosis

Except for the increased risk of ASCVD associated with dyslipidemia, emerging evidence suggests that elevated levels of LDL and TGs determine morpho-functional myocardial alterations. A Mendelian randomization study of 17,311 European subjects from the UK Biobank, who underwent both lipid profile assessment and cardiovascular magnetic resonance imaging, was recently performed by Aung et al. In this analysis, higher levels of TGs and LDL were associated with higher left ventricular mass, thus suggesting a direct, cytotoxic mechanism of action for lipids, with direct implications for cardiac structure and function [52]. Likewise, metabolic syndrome and, implicitly, hypertriglyceridemia favor the appearance of obstructive sleep apnea, which in turn is associated with increased left ventricular mass [53].

It appears that elevated levels of TGs, TRLs, and remnants are also linked to an increased risk of aortic valve stenosis. A cohort of 108,559 subjects from the Copenhagen General Population Study was included in another Mendelian randomization study, out of whom 1593 had an established diagnosis of aortic valve stenosis ensuing during a median follow-up time of 8.7 years. In the multifactorial adjusted analyses, higher concentrations of triglycerides and remnants were observationally and genetically linked to a higher rate of aortic valve stenosis. A novel etiopathogenic factor in aortic valve stenosis that warrants further research has been indicated by these findings [54].

Central arterial stiffness is a well-known marker of increased cardiovascular risk and mortality that can be reliably evaluated via the carotid-femoral pulse wave velocity. The Malmo Diet and Cancer study assessed the relationship between lipids and arterial stiffness on a cohort of 2505 patients during a median follow-up period of 17 years. It was indicated that TGs and HDL values predict carotid-femoral pulse wave velocity, whereas LDL does not [55].

On top of these findings, a growing body of evidence connects triglycerides and TRLs to diabetes and other metabolic diseases. At the same time, diabetes mellitus is linked to an increased risk of CVD and thromboembolic cerebrovascular events [56]. Advantageously, the triglyceride-to-HDL ratio is a surrogate not only of ASCVD but also of incident type 2 diabetes mellitus (T2DM). A longitudinal 12-year analysis of the Korean Genome and Epidemiology Study that selected 8655 patients aged 40 to 69 years with no evidence of T2DM documented that among the 1437 subjects who developed T2DM during the follow-up period, higher baseline values of the TGs-to-HDL ratio predicted the development of this metabolic disease. Importantly, this association was independent of other baseline markers of insulin resistance, such as the homeostasis model assessment of insulin resistance (HOMA-IR) [57].

Another metabolic index that is useful in predicting the risk of incident T2DM is the triglyceride-glucose index. A retrospective cohort study of 201,298 non-diabetic Chinese subjects followed up for 3.12 years proved that increased triglyceride-glucose index values independently predicted the risk of developing T2DM (hazard ratio 3.34, 95% confidence interval, 3.11–3.60). Significantly, this association was even more potent in women, young people (age <40 years), and in normal-weight and normotensive subjects [58].

Furthermore, in diabetic subjects, increased plasma levels of TGs and poor control of the TGs concentration with lipid-lowering drugs influence the decline of the estimated glomerular filtration rate (eGFR) and the onset of diabetic kidney disease (DKD). In a 3-year follow-up study that included 283 patients with new-onset T2DM, at the completion of the study, eGFR decline was higher among patients with poor control of body weight and LDL concentration, as well as in those with the highest elevation in TGs levels. Nevertheless, only TGs were significantly associated with the greatest reduction in eGFR values, and most importantly, the highest risk was registered in the subjects who experienced a transition from normal to increased TGs values and not in those with invariably increased TGs levels [59].

Starting from the premise that despite optimal control of serum glucose, blood pressure, and plasma LDL levels, there is a substantial residual risk of DKD in subjects with T2DM, a large retrospective observational 4-year follow-up study was conducted. A total of 15,362 diabetic subjects with an eGFR > 60 mL/min/1,73 m^2^, normoalbuminuria, and LDL ≤ 130 mg/dL were recruited in the final analysis. Abnormal TGs levels (TGs ≥ 150 mg/dL) independently increased the risk of DKD by 35%, while decreased concentrations of HDL enhanced the risk of DKD by 44%. These findings nominated TGs and HDL as independent risk factors for DKD, demanding proper therapeutic attention [60].

Finally, yet importantly, in diabetic subjects, the increased risk of adverse cardiovascular events seems to be significantly modulated by hypertriglyceridemia. A recent analysis of the Action of Health in Diabetes (Look AHEAD) study comprising 4199 individuals emphasized that in overweight and obese diabetic patients, metabolic dyslipidemia, characterized by increased levels of TGs and decreased plasma concentration of HDL, is associated with a higher risk of atherosclerotic cardiovascular events [61]. Specifically, in middle-aged, non-insulin-treated diabetic men, lower adiponectin concentrations are connected to increased production of plasma thrombin, and plasma TGs substantially influence this association. Of note, decreased concentration of adiponectin and enhanced generation of plasma thrombin independently increase CVD risk [62]. Thrombin, by favoring clot formation, is linked to an increased risk of thromboembolic events [63].

## 6. New Therapeutic Perspectives for Hypertriglyceridemia

In the past decades, there have been significant advances in the therapeutic approach to dyslipidemias. Statins persist in being the mainstay therapy for hyperlipidemia and ASCVD, able to reduce plasma TGs concentration by 20% [28,64]. However, a broad range of lipid-lowering agents targeting triglycerides and apoB has recently come to light as beneficial in supplementarily decreasing cardiovascular mortality beyond that achieved via LDL reduction [65]. Besides, in patients with ASCVD, lifestyle interventions exert complementary hypotriglyceridemic actions.

Fibrates are deemed the most powerful therapeutic drugs for managing hypertriglyceridemia, but their potency to reduce overall cardiovascular risk is modest. Pemafibrate is a selective modulator of peroxisomal proliferator-activated receptor-α that has lately received a particular focus due to its favorable side-effect profile [66].

Several phase 2 and phase 3 trials have demonstrated the efficacy and safety of pemafibrate as well as its non-inferiority to other fibrates. The PemafibRate study tO Validate a 52-week efficacy and safety In patients with type 2 Diabetes comorbid with Elevated triglyceride levels (PROVIDE study) was designed to evaluate the efficacy and safety of pemafibrate versus placebo when administrated in diabetic patients with hypertriglyceridemia. In treatment period 1, subjects were assigned to either pemafibrate at a dose of 0.2 or 0.4 mg/d or placebo for 24 weeks. Following the completion of this first period of treatment, placebo was switched to pemafibrate at a dose of 0.2 mg and continued unchanged in the treatment group up to week 52. Treatment with pemafibrate resulted in a significant fall in TGs levels and TRLs concentration (percentage changes in TGs levels of −48.2% in the placebo/pemafibrate group consisting of 57 patients, −42.3% in the 0.2 mg/d of pemafibrate group including 54 individuals, and −46.4% in the pemafibrate 0.4 mg/day study group comprising another 55 diabetic subjects) [67].

The non-inferiority of pemafibrate to fenofibrate was ascertained by another phase 3 trial that included 527 subjects. Nevertheless, the most important finding of this randomized study was the favorable benefit-risk balance of pemafibrate versus fenofibrate; the administration of this triglyceride-lowering agent resulted in fewer adverse events on kidney/liver function and a more favorable safety profile when compared to fenofibrate [68].

Concerning ezetimibe’s action on hypertriglyceridemia, a systematic review and meta-analysis of randomized controlled trial reported only a slight decrease in triglyceride levels (−8.06%, 95% CI: −10.92 to −5.2) with this drug. Ezetimibe inhibits the intestinal absorption of dietary and biliary cholesterol, thus being more powerful in reducing total and LDL-cholesterol than TGs levels [69].

The hypotriglyceridemic action of omega-3 fatty acids has been known for many years. However, the revival of its cardiovascular benefits came with the results of the Reduction of Cardiovascular Events with Icosapent ethyl- Intervention Trial (REDUCE-IT), that demonstrated a 25% relative risk reduction in adverse cardiovascular events with icosapent ethyl, both in primary (diabetic subjects) and secondary prevention [70,71].

The REDUCE-IT trial was a multicenter, randomized, double-blind, placebo-controlled trial that evaluated the effects of 2 g of icosapent ethyl twice daily among subjects at high risk for ASCVD, with elevated concentrations of triglycerides, but with steady levels of LDL under pharmacological therapy with statins. The results of this trial demonstrated that the daily administration of 4 g of icosapent ethyl per total significantly decreases the risk of ischemic events, including cardiovascular death [72]. On this basis, it was assumed that almost 3 million adults from the United States of America are qualified for icosapent ehtyl treatment and that over 70,000 of the total ASCVD events could be prevented [73].

Prior to REDUCE-IT, two major randomized, placebo-controlled trials failed to prove the benefits of omega-3 fatty acids when employed in primary prevention. A Study of Cardiovascular Events in Diabetes (ASCEND study) with *n*-3 fatty acids randomized 15,480 diabetic subjects with no evidence of ASCVD to either 1g of omega-3 fatty acids daily or placebo and documented no significant difference in the risk of serious adverse cardiac events with omega-3 fatty acids [74].

VITAL (Vitamin D and Omega-3 Trial) was the other randomized, placebo-controlled trial that sought to assess the efficacy of *n*-3 fatty acids in the primary prevention of CVD as well as cancer among men aged 50 years and above and women aged 55 years and above. It comprised a total of 25,871 participants who were randomly assigned to either 1 g per day of omega-3 fatty acids or placebo and followed up for 5.3 years. No reduction in the number of major cardiovascular events or cancer in the general population was recorded this time, either [75].

On top of the triglycerides-lowering action, omega-3 polyunsaturated acids appear to exert a beneficial effect on endothelial function. In a single-center, prospective interventional study, 47 individuals with mild hypertriglyceridemia and no evidence of CVD were assigned to 1800 mg daily of eicosapentaenoic acid for 6 months, while another 44 age- and sex-matched subjects formed the control group. TGs and flow-mediated dilation were evaluated at baseline and at the completion of the 6-month treatment. Eicosapentaenoic acid produced a decrease in total TGs levels and an increase in flow-mediated dilation, an increase that was dependent on baseline HDL concentration and on the change in eicosapentaenoic acid/arachidonic acid ratio [76].

The identification of proprotein convertase subtilisin/kexin type 9 (PCSK9) inhibitors revolutionized the therapeutic management of dyslipidemia. Apart from a substantial reduction in LDL and lipoprotein(a) [Lp(a)] levels, two well-known risk factors for ASCVD, the inhibition of PCSK9 with evolocumab appears effective in decreasing VLDL plasma concentrations. Additionally, the magnitude of this reduction seems to be directly dependent on the baseline levels of Lp(a). Zhang et al. performed a nuclear magnetic resonance metabolomic profiling of 30 patients with increased concentrations of Lp(a) that were randomized to either 420 mg of evolocumab every 4 weeks or placebo. Following 16 weeks of treatment, PCSK9 inhibition resulted in a marked decrease of VLDL, ILDL, and LDL levels and a considerable reduction of Lp(a) concentrations. Notably, higher values of baseline Lp(a) were linked to a more pronounced reduction of VLDL particles [77].

In contrast, Taskinen et al. failed to prove a significant influence on TRLs concentrations (mostly chylomicrons and VLDL_1_) and, therefore, on plasma levels of apoB48 and apoB100 following the administration of 140 mg of evolocumab every two weeks for 12 weeks. However, in their study population, consisting of 13 diabetic subjects, PCSK9 inhibition led to a substantial decrease of VLDL_2_, IDL, and LDL, important cholesterol-carrying particles [78].

As already discussed, the inhibition of ANGPTL3 and apoCIII are other promising therapeutic approaches to hypertriglyceridemia targeting these proteins with ribonucleic acid (RNA) interfering therapies are presently being exploited [16,79]. Volanesorsen is a second-generation chimeric antisense therapeutic oligonucleotide that produces a dose-dependent decrease in plasma apoCIII levels and, therefore, in TGs concentration, and is currently undergoing licensing [80].

In a phase 3, double-blind, randomized trial, including 66 subjects with familial chylomicronemia syndrome assigned to either volanesorsen or placebo in a 1:1 ratio, the 3-month treatment with volanesorsen resulted in a 77% decrease in mean TGs concentrations. The most frequently reported side effects in the volanesorsen group were thrombocytopenia and injection-site reactions [81].

Another small interfering RNA agent, inclisiran, appears to be effective in decreasing TGs and apolipoprotein B concentrations in patients with familal hypercholesterolemia, as emphasized by the results of the ORION-9 trial [82]. Inclisiran acts by inhibiting the hepatic synthesis of PCSK9. In ORION-10 and ORION-11 trials, it proved to be beneficial in decreasing LDL levels by 50% when delivered subcutaneously every 6 months over a period of 18 months in patients with ASCVD or ASCVD risk equivalent and elevated concentrations of LDL despite statin-treatment. Importantly, in these two trials, inclisiran also decreased the plasmatic levels of TGs [83]. Thus, inclisiran shows promising results for the treatment of hypertriglyceridemia.

Except for the conventional TGs-lowering drugs, several nontraditional hypolipemiant pharmacological agents demonstrated important hypotriglyceridemic actions. In subjects with T2DM, liraglutide, a glucagon-like peptide 1 (GLP-1) receptor agonist, seems to exert a beneficial, lowering effect on TRLs. The action of liraglutide on postprandial apoB48 and apoB100 metabolism has been explored in a study where 14 diabetic subjects were assigned to a 16-week treatment with 1.8 mg/day of liraglutide and 4 to placebo. After a single fat-rich meal, the GLP-1 agonist produced a significant decrease in apoB48 synthesis in CMs and increased the TGs/apo48 ratio of CMs. Additionally, CMs clearance increased by 90% in the group treated with liraglutide, and VLDL_1_ secretion significantly decreased. This finding suggests that in diabetic patients, the treatment with GLP-1 agonists can result in decreased production of atherogenic remnants, thus creating the setting for further research [84].

In diabetic subjects, it appears that not only GLP-1 agonists but also sodium/glucose cotransporter 2 (SGLT2) inhibitors exert beneficial effects on the lipid profile. In a study that included 50 diabetic patients assigned to 10 mg daily of empagliflozin for a 6-month treatment, a significant decrease in fasting and post-prandial TGs concentration was recorded. Additionally, the reduction in TGs concentration was the strongest predictor for the flow-mediated dilatation improvement and, therefore, for endothelial function recovery [85]. In Table 1, we have summarized all the above-mentioned triglyceride-lowering agents, including the traditional hypotriglyceridemic drugs with established benefits and the unconventional hypolipemiant medication with potential advantageous action on TGs and TRLs profile.

## 7. Conclusions

Premised on the fact that, on statin-treated subjects, there is a considerable residual risk of cardiovascular events, research has focused on the putative role of triglycerides, triglyceride-rich lipoproteins, and remnants on atherosclerosis. Several epidemiological population-based and Mendelian randomization studies linked TRLs and remnants to ASCVD, but there is still insufficient evidence in this field. With the triglyceride-glucose index and triglyceride to high-density lipoprotein ratio emerging as surrogate indicators of peripheral artery disease and atherosclerotic cerebrovascular disease, one can conclude that further research addressing the intricate relationship between triglycerides and atherosclerosis is warranted. On top of the triglycerides-mediated ASCVD, exposure to high concentrations of TGs has been associated with adverse cardiac remodeling, aortic valve stenosis, and incident type 2 diabetes mellitus. Therefore, the importance of targeting hypertriglyceridemia with both lifestyle changes and pharmacotherapy is being emphasized. With regard to triglyceride-lowering pharmacological agents, we have witnessed major advances in the last few years, with the emergence of RNA interfering therapies and the revival of omega-3 fatty acids.

## Figures and Tables

**Table 1:** Triglycerides-lowering pharmacological agents.

Drug	Action on TGs Levels
Statins	Lower TGs levels by 20% [18,54] First-line therapy
Fibrates (e.g., pemafibrate)	The most potent drugs in managing hypertriglyceridemia Produce a decrease of up to 50% in TGs concentrations [58] Pemafibrate is non-inferior to other fibrates and has a more favorable safety profile Their potency to decrease overall cardiovascular risk is modest
Omega 3 fatty acids (e.g., Icosapent Ethyl)	Demonstrated a 25% relative risk reduction in adverse cardiovascular events both in primary and secondary prevention when administrated at a high dose [63] Exert a beneficial effect on endothelial function assessed via flow-mediated dilation [66]
Ezetimibe	Produces only a slight decrease in TGs levels [59]
PCSK9 inhibitors (e.g., evolocumab)	Controversial data Reduction in VLDL, IDL, LDL, and Lp(a) levels The decrease in VLDL levels is dependent on baseline Lp(a) values [67] The lowering effect is more pronounced on VLDL_2_ levels than on VLDL_1_ [68]
Volanesorsen	A second-generation chimeric antisense therapeutic oligonucleotide that decreases plasma apoCIII and TGs levels in a dose-dependent manner [71]
Inclisiran	Small interfering RNA agent Majorly effective in reducing LDL concentration but also lowered TGs levels in ORION 9, 10, and 11 trials [72,73]
GLP-1 receptor agonists (e.g., liraglutide)	Decreases apoB48 synthesis in CMs Apparently decreased production of atherogenic remnants in diabetic patients [74]
SGLT2 inhibitors (e.g., empagliflozin)	Decrease in fasting and post-prandial TGs concentration and a flow-mediated dilation improvement [75]

TG: triglyceride; VLDL: very-low-density lipoproteins; IDL: intermediate-density lipoprotein; Lp(a): lipoprotein(a); RNA: ribonucleic acid; CMs: chylomicrons.

## Data Availability

Not applicable.
